# Risk Factors and Preventive Measures for Cardiovascular Diseases

**DOI:** 10.3390/jcm13113308

**Published:** 2024-06-04

**Authors:** Katharina Preisner, Svetlana Hetjens

**Affiliations:** Department of Medical Statistics, Center for Preventive Medicine and Digital Health, Medical Faculty Mannheim, University of Heidelberg, Theodor-Kutzer-Ufer 1-3, D-68167 Mannheim, Germany

**Keywords:** prevention, cardiovascular diseases, mortality

## Abstract

**Background:** Cardiovascular diseases are the most frequent cause of death worldwide. The aim of this study was to identify and demonstrate correlations between mortality data and etiological factors in EU countries. The relationships presented could thus provide a better understanding of etiological factors and possible points for interventions to prevent cardiovascular diseases. The focus was on the following diseases: hypertensive heart disease, atrial flutter/atrial fibrillation, myocardial infarction, and ischemic heart disease, as well as heart failure. **Methods:** The data in this study come from WHO databases. Connections between the mortality rates and the possible influencing factors were analyzed. The significant factors from the correlation analysis were simultaneously evaluated using a stepwise multiple regression analysis. **Results:** Analysis of hypertensive heart disease in women reveals the following factors to be significant: drug expenses, health expenses on gross domestic product, and smoking. For men, population density, first admission to a drug treatment center, and drug expenses per person emerged as important factors. Admission to drug treatment centers and length of hospitalization were significant factors for atrial fibrillation and flutter. Fine dust pollution was the most important factor in heart failure. The most important influencing factor for myocardial infarction and ischemic heart disease is nitrogen dioxide concentration. For women, the second highest value is health expenses, followed by the number of outpatient contacts per year. For men, outpatient contacts are in second place. **Conclusions:** Prevention measures must be taken by the government. The extent to which population density has an influence on cardiovascular diseases should be examined in more detail. In order to reduce the number of initial admissions to drug treatment centers, behavioral prevention related to drug use could be improved.

## 1. Introduction

According to the World Health Organization (WHO), an estimated 17.9 million people worldwide die from cardiovascular disease every year. That is 32% of all deaths worldwide [1]. Also, in Europe and the European Union (EU), most people die every year as a result of cardiovascular disease (international: “cardiovascular disease” (CVD)). According to the European cardiovascular disease statistics for 2019, the number of deaths due to cardiovascular disease across Europe is 3.9 million, which corresponds to 45% of total deaths. Within the EU, there are 1.8 million, or 37%, of the total deaths [2].

The registry of cardiovascular disease is carried out according to the ICD-10 classification, which is recognized worldwide. ICD stands for “International Statistical Classification of Diseases and Related Health Problems” [3]. The following four most frequent cardiovascular diseases were included in this study [4]: I11 hypertensive heart disease, I48 atrial flutter and fibrillation, I50 heart failure, and I21-I25 myocardial infarction (I21. Acute myocardial infarction; I22. Recurrent myocardial infarction; I23. Certain acute complications after acute myocardial infarction; I24. Other acute ischemic heart disease; and I25. Chronic ischemic heart disease). Categories I21 to I25 are combined, as the different coding habits of myocardial infarction may vary from country to country.

According to the World Health Organization (WHO), 80% of cardiovascular deaths could be avoided by preventive measures [1].

The frequency and occurrence of cardiovascular diseases are determined by multiple factors. This makes the prevention of cardiovascular diseases a cross-sectional task that has many possible approaches. For example, the “Health 2020” plan was initiated by the World Health Organization to improve health and prevention measures [5].

Within cardiovascular risk factors, a distinction is made between risk factors that can be influenced by interventions and measures and risk factors that cannot be influenced by these measures. The most important risk factors for which a causal relationship with the occurrence of cardiovascular events has been proven in several studies are:

- Nicotine abuse.
- Arterial hypertension (regular blood pressure values at rest ≥ 140/90 mmHg).
- Hypercholesterolemia (total cholesterol > 240 mg/dL and LDL cholesterol values of ≥160 mg/dL).

Furthermore, cardiovascular risk factors include diabetes mellitus (HbA1c > 6.5%), impaired glucose tolerance (Nü BG ≥ 100 mg/dL), physical inactivity, and obesity. These also include hypertriglyceridemia, an increased homocysteine level, an increase in the Lp(a) value, and increased C-reactive protein (CRP) levels, as well as depression.

Risk factors that cannot be influenced are age (m ≥ 45 and w ≥ 55), male gender, and positive family history (coronary heart disease (CHD)/heart attacks in first-degree family members before the age of 55 in men and 65 in women) [6,7,8].

The aim of this study was to identify and demonstrate correlations between mortality data and etiological factors in EU countries. The relationships presented could thus provide a better understanding of etiological factors and possible points for interventions to prevent cardiovascular diseases and ultimately provide a hint to reduce disease-specific mortality.

## 2. Material and Methods

**Figure 1:**
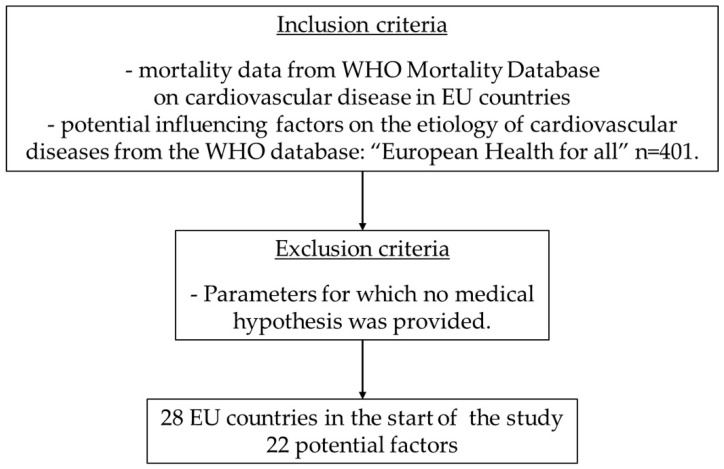
Inclusion and exclusion criteria.

The etiology of cardiovascular disease is multifactorial. The medical and individual risk factors of arteriosclerosis, as one of the most important causes of cardiovascular diseases, have been investigated in many studies. In order to obtain a comprehensive picture of the factors influencing the etiology within the EU countries, this study not only analyzed some classic medical risk factors for cardiovascular diseases but also the facts and living conditions of the respective countries as well as personal living conditions, such as alcohol consumption or people’s literacy rates (Figure 1).

Potential influencing factors on the etiology of cardiovascular diseases were selected from the groups of social, health, and environmental factors and individual behavior. The data used in this study are from the WHO database titled “European Health for all”.

1. Social factors:
  - Average population density (km^2^).
  - Unemployment rate in percent (%).
  - Gross domestic product per person (GDP $ (p.p.)).
  - Gini-coefficient as a measure of inequality in income distribution.
  - Literacy rate of the population older than 15 years (%).
  - Health expenses as a percentage of gross domestic product (%).
  - Health expenses in dollars per person.
2. Health factors:
  - Number of hospitals per 100,000 inhabitants.
  - Number of doctors per 100,000 inhabitants.
  - Average length of hospitalization.
  - Number of outpatient contacts per person/year.
  - Drug expenses/person.
3. Environmental factors:
  - Average annual sulfur dioxide concentration (SO_2_) in the capital (μg/m^3^).
  - Average annual fine dust pollution < 10 µm (PM_10_) in the capital (μg/m^3^).
  - Average annual nitrogen dioxide concentration (NO_2_) in the capital (μg/m^3^).
  - Average annual ozone concentration (O_3_) in the capital (μg/m^3^).
4. Individual behavior:
  - Number of regular daily smokers (tobacco consumption) older than 15 years (%).
  - Number of people suffering from diabetes mellitus per 100,000.
  - Age-standardized prevalence of overweight (BMI > 25 kg/m^2^) from the age of 18, WHO estimate (%).
  - Pure alcohol consumption per person/per liter from the age of 15.
  - First admission to a drug treatment center per 100,000.
  - Amount of private health costs in total health expenses, out-of-pocket spending (%).

The data for the period from 2010 to 2015 were analyzed. Age standardization of the mortality data was carried out to exclude the possibility that differences in mortality between these countries were due to different age structures. Germany was used as the standard population. The correlation analysis was carried out to determine the relationships between the selected influencing variables and the mortality rates. A multiple stepwise regression analysis was then carried out on the significant factors in the correlation analysis in order to evaluate several influencing variables simultaneously. The stepwise regression analysis provides a test variable (F) according to which the *p*-value is calculated. The larger this value, the more important the parameter is in the model and, therefore, the most important factor with the most influence on the dependent parameter (mortality rate). Multiple imputation (a missing data procedure) was used for this regression analysis. The statistical significance level was considered to be significant at a *p* value of <0.05. The evaluation was carried out using the SAS 9.3 software.

## 3. Results

### 3.1. Analysis of Hypertensive Heart Disease

For both sexes, the following factors were significant in the correlation analysis: population density, gross domestic product, Gini-coefficient, regular smokers, first admission to a drug treatment center, nitrogen dioxide concentration, ozone concentration, health expenses in gross domestic product and per person, and pharmaceutical expenses.

The stepwise regression and the resulting test variable show that for women, pharmaceutical expenses per person are the most important factor, followed by health expenses in gross domestic product and smoking (Table 1).

For men, the most important factor is population density, followed by first admissions to a drug treatment center per 100,000 and drug expenses per person (Table 2).

### 3.2. Analysis of Atrial Flutter and Atrial Fibrillation

The following factors are significant for both genders:

Unemployment rate, gross domestic product, Gini-coefficient, smokers, obesity, alcohol consumption, first admissions to a drug treatment center, hospitals per 100,000 inhabitants, doctors per 100,000, average length of hospitalization, outpatient contacts, health expenses in GDP and per person, and private health expenses.

The stepwise regression and the resulting test variable show that first admissions to drug treatment centers and the average length of hospitalization are important and significant factors for both genders (Table 3 and Table 4).

### 3.3. Analysis of Heart Failure

The women’s correlation analysis revealed significant values for the factors gross domestic product, sulfur dioxide concentration, fine dust concentration, average length of hospitalization, and private health expenses. The value of health expenses per person is of low significance (Table 5).

The men’s correlation analyses revealed significant values for the factors gross domestic product, Gini-coefficient, sulfur dioxide concentration, fine dust concentration, average length of hospitalization, and private health expenses. The value of health expenses in GDP and per person, as well as private health expenses, is of low significance (Table 6).

### 3.4. Analysis of Acute Myocardial Infarction/Ischemic Heart Disease

Significant results for women can be seen for GDP, literacy rate, smoking, alcohol, first admission to a drug treatment center, nitrogen dioxide concentration, length of hospitalization, outpatient contacts, health expenses in gross domestic product and per person, as well as for private health expenses.

Analyzing men, significant values emerge for the following factors: unemployment rate, gross domestic product, Gini-coefficient, literacy rate, smokers, alcohol, first admission to a drug treatment center, nitrogen dioxide concentration, length of hospitalization, outpatient contacts, health expenses in gross domestic product and per person, as well as private health expenses. Population density was weakly significant.

The stepwise regression and the resulting test variable (*F*) show that the nitrogen dioxide concentration, health expenses per person, and outpatient contacts are the most significant factors for women (Table 7).

For men, too, the most important factors are the nitrogen dioxide concentration and outpatient contacts (Table 8).

## 4. Discussion

Cardiovascular diseases are the most common cause of death in industrialized nations [7]. Despite declining mortality rates in this area in some places, no other disease kills more people every year. Studies and analyses on CVD are important because they can lead to measures and approaches for prevention.

This study focused on EU countries in relation to cardiovascular disease. Determinants and relationships from environmental and socioeconomic factors are taken into account and placed in context with CVD mortality and possible interventions for the prevention of cardiovascular diseases. The results could reveal new possibilities for intervention and prevention of cardiovascular diseases and thus bring about a long-term reduction in mortality from cardiovascular diseases. The analysis carried out here could also be useful in long-term studies.

The data in this study come from WHO databases. The mortality rates and factors were analyzed for the years 2010 to 2015. The period of five years is limited, so only trends can be described in terms of the direction development is going. However, this period is too short to make a statement about lasting developments and changes, as factors such as smoking and tobacco consumption have long-term effects. There is also the following limitation: The missing values in the regression analysis were supplemented using multiple imputations, as this was the only way to analyze all countries and factors. However, multiple imputations are only an estimated value.

Cardiovascular diseases are caused by multiple factors. For example, by personal factors such as gender and age, but also by lifestyle, social environment, access to medical care, and economic and social conditions. Measurements to prevent cardiovascular diseases can apply to any of these.

A possible approach to comparing prevention measures between EU countries is the use of international guidelines. The guidelines for cardiovascular diseases are published by the European Society of Cardiology (ESC) [9]. In addition, specific prevention measures are described in the guidelines for clinical disease patterns. The EUROASPIRE IV study showed that the preventive measures recommended are rarely used [10,11]. In total, a direct and objective survey of the use of preventive measures at the individual level is not easily realizable. For example, one suggestion from the guidelines for the prevention of cardiovascular disease is to control the number of participants in withdrawal programs and to advise on physical activity or nutrition. Observing the long-term application of preventive measures as part of a long-term prospective cohort study such as the Framingham sample could be one possible way to observe lifestyle factors. Up to now, there is only feedback on whether the guidelines are generally followed. Establishing more detailed feedback would be a possibility for comparison, e.g., whether there are instructions for procedures or checklists for diagnostics and therapy in hospitals. Each hospital, as well as the corresponding cardiological society, could then provide feedback as to the extent to which the guideline is being applied nationwide; this information should be given in relative terms. Cardiovascular imaging is an important part of accurately categorizing individuals according to their risk profiles, allowing precise therapeutic strategies to be developed for patients at increased cardiovascular risk [12].

Expenditure on medicines and health in women were important factors related to women’s lower economic independence, which is a cardiovascular risk factor [13]. In the individual disease groups, admission to drug treatment centers and, thus, drug consumption were the most important factors for atrial fibrillation and flutter. Prevention programs at the government level should be initiated to intervene here. Fine dust pollution was found to be the main factor in heart failure. Prevention measures must also be initiated at the government level, such as measures to comply with the legally prescribed levels of fine dust pollution. Nitrogen dioxide concentration was found to be the most important factor in ischemic heart disease and myocardial infarction. Efforts must be made to improve air pollution. After inhalation, particulate matter (PM_2.5_) is absorbed via the lung tissue into the blood vessels, leading to atherosclerotic changes [14]. Prevention of air pollution also needs to be initiated at the government level. For hypertension, drug expenditure was the most important factor. Prevention here lies primarily in medical treatment. One potential measure would be consistent implementation of the guidelines.

Future prevention measures lie particularly in the cardiovascular imaging of accurately categorizing individuals according to their risk profiles, the economic independence of women, as well as good local medical care, behavioral prevention related to drugs, and the control of air pollution.

## 5. Conclusions

Prevention measures must be taken by the government. The extent to which population density has an influence on cardiovascular diseases should be examined in more detail. In order to reduce the number of initial admissions to drug treatment centers, behavioral prevention related to drug use could be improved.

## Figures and Tables

**Table 1:** Multiple stepwise regression for hypertensive heart disease in women (*R*^2^ = 0.6344).

Parameter	*p*-Value	*F*-Value
Nitrogen dioxide concentration (μg/m^3^)	0.0009	11.58
Gini-coefficient	0.0003	13.65
Ozone concentration (μg/m^3^)	0.0001	15.66
Population density (km^2^)	<0.0001	27.51
Health expenses ($ p.p.)	<0.0001	45.11
First admission to a drug treatment center per 100.000	<0.0001	62.15
Regular smokers (%)	<0.0001	74.09
Health expenses (GDP %)	<0.0001	78.99
Drug expenses ($ p.p.)	<0.0001	110.30

**Table 2:** Multiple stepwise regression for hypertensive heart disease in men (*R*^2^ = 0.6189).

Parameter	*p*-Value	*F*-Value
Gini-coefficient	0.0406	4.28
Health expenses ($ p.p.)	<0.0001	24.06
Nitrogen dioxide concentration (μg/m^3^)	<0.0001	25.55
Health expenses (GDP %)	<0.0001	27.42
Regular smokers (%)	<0.0001	53.24
First admissions to a drug treatment center per 100,000	<0.0001	58.71
Drug expenses ($ p.p.)	<0.0001	58.98
Population density (km^2^)	<0.0001	62.39

**Table 3:** Multiple stepwise regression for atrial flutter and atrial fibrillation in women (*R*^2^ = 0.9359).

Parameter	*p*-Value	*F*-Value
Gini-coefficient	0.0002	14.61
GDP ($ p.p.)	0.0002	15.04
Overweight (%)	<0.0001	25.01
Alcohol consumption per liter	<0.0001	25.37
Health expenses ($ p.p.)	<0.0001	31.07
Health expenses (GDP %)	<0.0001	33.60
Regular smokers (%)	<0.0001	143.84
Hospitals per 100,000 inhabitants	<0.0001	166.17
Average length of hospitalization	<0.0001	385.58
First admissions to drug treatment centers per 100,000	<0.0001	391.06

**Table 4:** Multiple stepwise regression for atrial flutter and atrial fibrillation in men (*R*^2^ = 0.8738).

Parameter	*p*-Value	*F*-Value
Outpatient contacts per person per year	0.0366	4.46
Doctors per 100,000	0.0238	5.23
Alcohol consumpton per liter	0.0004	13.43
Health expenses ($ p.p.)	<0.0001	44.51
Out-of-pocket of total health expenses (%)	<0.0001	53.82
Overweight (%)	<0.0001	57.28
Hospitals per 100,000 inhabitants	<0.0001	58.47
Regular smokers (%)	<0.0001	93.38
Average duration of hospitalization	<0.0001	153.87
First admission to drug treatment centers per 100,000	<0.0001	169.61

**Table 5:** Multiple stepwise regression for heart failure in women (*R*^2^ = 0.6289).

Parameter	*p*-Value	*F*-Value
Sulfur dioxide concentration (μg/m^3^)	0.0408	4.27
GDP ($ p.p.)	0.0236	5.25
Out-of-pocket of total healthcare expenses (%)	0.0040	8.55
Average duration of hospitalization	0.0016	10.43
Fine dust pollution (μg/m^3^)	<0.0001	139.79

**Table 6:** Multiple stepwise regression for heart failure in men (*R*^2^ = 0.6919).

Parameter	*p*-Value	*F*-Value
GDP ($ p.p.)	<0.0001	32.04
Health expenses ($ p.p.)	<0.0001	32.24
Sulfur dioxide concentration (μg/m^3^)	<0.0001	40.73
Average duration of hospitalization	<0.0001	54.96
Fine dust pollution (μg/m^3^)	<0.0001	109.83

**Table 7:** Multiple stepwise regression for acute myocardial infarction/ischemic heart disease in women (*R*^2^ = 0.8583).

Parameter	*p*-Value	*F*-Value
Out-of-pocket of total health expenses (%)	<0.0001	6.60
Literacy rate (%)	<0.0001	17.10
Health expenses (GDP %)	<0.0001	22.29
Alcohol consumption per liter	<0.0001	22.73
Unemployment rate (%)	<0.0001	54.50
First admission to drug treatment centers per 100,000	<0.0001	67.29
Gini-coefficient	<0.0001	70.97
Regular smokers (%)	<0.0001	76.43
Outpatient contacts per year	<0.0001	85.04
Health expenses ($ p.p.)	<0.0001	86.47
Sulfur dioxide concentration (ug/m^3^)	<0.0001	164.16

**Table 8:** Multiple stepwise regression for acute myocardial infarction/ischemic heart disease in men (*R*^2^ = 0.8318).

Parameter	*p*-Value	*F*-Value
Health expenses (GDP %)	0.1162	2.50
Gini-coefficient	0.0127	6.39
Health expenses ($ p.p.)	<0.0001	17.79
Out-of-pocket of total healthcare expenses (%)	<0.0001	21.34
Regular smokers (%)	<0.0001	29.40
First admission to drug treatment centers per 100,000	<0.0001	38.55
GDP ($ p.p.)	<0.0001	41.26
Average duration of hospitalization	<0.0001	45.85
Outpatient contacts per person per year	<0.0001	71.57
Nitrogen dioxide concentration (ug/m^3^)	<0.0001	113.34

## Data Availability

The data presented in this study are available upon request from the corresponding author.
